# Prolapsus Uteri

**Published:** 1871-11

**Authors:** 


*Prolapsus Uteri*; its chief causes and Treatment. By Thomas Addis Emmett, M.D.

This is a very valuable paper, and is worthy careful perusal by the profession. The chief causes are grouped under four heads, it may result from one of these, or several combined. "First, the uterus may be crowded down into the pelvis from abnormal pressure above, by the weight of some extraneous development in the cavity, from habitual constipation, tight lacing, &c. Second, the prolapse resulting from increased size of the organ, from imperfect involution, obstructed circulation in the organ itself, or elsewhere, from pathological changes of its tissues, the effect of inflammation, or from the development of tumors intimately connected. Third, after parturition, the vagina may remain dilated with a loss of tone in part or throughout the canal. Fourth, from laceration of the perineum, we find rectocele and cystocele resulting, and with the prolapsing vaginal walls, detect the descending uterus at various points, from a slight displacement to complete procidentia." For treatment he recommends restoration of the health of the patient to a certain standard before instituting the local treatment. For the latter he advises large hot-water injections, a pint or two at 98° or 100° F, thrown into the vagina This he has practiced for the past nine years and has found it very efficacious. Other plans of treatment are mentioned. Cases which cannot be treated in any other way, are selected for surgical interference.

